# Exploring the Overestimation of Conjunctive Probabilities

**DOI:** 10.3389/fpsyg.2013.00101

**Published:** 2013-03-04

**Authors:** Håkan Nilsson, Jörg Rieskamp, Mirjam A. Jenny

**Affiliations:** ^1^Department of Psychology, University of BaselBasel, Switzerland; ^2^Department of Psychology, Uppsala UniversityUppsala, Sweden

**Keywords:** judgment, accuracy, conjunctive probability, overestimation, conjunction fallacy, configural weighted average hypothesis

## Abstract

People often overestimate probabilities of conjunctive events. The authors explored whether the accuracy of conjunctive probability estimates can be improved by increased experience with relevant constituent events and by using memory aids. The first experiment showed that increased experience with constituent events increased the correlation between the estimated and the objective conjunctive probabilities, but that it did not reduce overestimation of conjunctive probabilities. The second experiment showed that reducing cognitive load with memory aids for the constituent probabilities led to improved estimates of the conjunctive probabilities and to decreased overestimation of conjunctive probabilities. To explain the cognitive process underlying people’s probability estimates, the configural weighted average model was tested against the normative multiplicative model. The configural weighted average model generates conjunctive probabilities that systematically overestimate objective probabilities although the generated probabilities still correlate strongly with the objective probabilities. For the majority of participants this model was better than the multiplicative model in predicting the probability estimates. However, when memory aids were provided, the predictive accuracy of the multiplicative model increased. In sum, memory tools can improve people’s conjunctive probability estimates.

## Exploring the Overestimation of Conjunctive Probabilities

Over the last decades, hundreds of studies have explored people’s ability to estimate conjunctive probabilities (Wedell and Moro, 2008). This vast literature teaches us that if a group of people are shown a description of a woman who resembles the prototypical feminist and are then asked to estimate the probability that this woman is a bank teller and the probability that she is a feminist bank teller, the modal response is likely to be that the latter probability is higher than the former (a phenomenon known as the conjunction error; Tversky and Kahneman, 1983). The literature also teaches us that the conjunction error can be observed in a wide range of populations and with a wide range of tasks (e.g., Crandall and Greenfield, 1986; Davidson, 1995; Hertwig and Chase, 1998; Wedell and Moro, 2008; Nilsson et al., 2009). Oddly, there is one aspect about which the literature is silent: it tells us nothing about how well conjunctive probability estimates generated by humans correspond to conjunctive probabilities in the environment. Thus, in some sense, the literature tells us nothing about people’s ability to estimate *accurate* conjunctive probabilities.

We explored the accuracy of conjunctive probability estimates and factors that can potentially affect this accuracy. As emphasized by Hammond (1996; see also Dunwoody, 2009), the accuracy of a judgment, such as a probability judgment, can be evaluated in at least two ways: by whether it corresponds with facts about the environment (*the correspondence criterion*) or by whether it coheres with standards set by normative theories (*the coherence criterion*). A problematic aspect of previous literature on the accuracy of conjunctive probability estimates is that almost all attention has been devoted to the coherence criterion. We fear that this disproportionate focus might have led to an overly negative view of people’s ability to estimate conjunctive probabilities. Therefore, to get a more complete picture of people’s ability to estimate accurate conjunctive probabilities, we conducted two experiments where we focused on both coherence and correspondence. The paper begins with a discussion of what a probability is and how its accuracy can be measured. This discussion is followed by a review of the literature on conjunctive probability estimation, which leads into the presentation of the two experiments.

What is a probability? In this article, we adopt the frequentist interpretation of the term probability (see Hacking, 1975), according to which probabilities can be assessed fairly accurately by observing the occurrence of events in the environment. For example, imagine two boxes, A and B, both filled with marbles that are either black or white. The distribution of marbles in these boxes can be estimated by repeatedly sampling from both boxes. The probability of, for example, sampling a black marble from Box A can then be estimated from the frequency of black marbles sampled from Box A. Suppose Box A contains 50% black and 50% white marbles, whereas Box B contains 80% black and 20% white marbles. In this case, the constituent probability of sampling a black marble in a single draw equals 0.50 for A and 0.80 for B. To assess the probability of conjunctively sampling a black marble out of Box A *and* a black marble out of Box B, probability theory prescribes multiplying the two constituent probabilities:

(1) $$p\left(\text{A\&B}\right)=p\left(\text{A}\right)\times \;p\left(\text{B}\right)$$

This multiplicative rule holds whenever the two constituents are independent, as in the experiments presented below. Thus, when drawing one marble from Box A and one from Box B, the probability of sampling two black marbles is 0.50 × 0.80 = 0.40. In the following we frequently refer to the term *objective probability*, which is a probability computed with perfect knowledge of the environment and, in the case of conjunctive probabilities, according to Eq. 1.

What determines the accuracy of a probability estimate? According to the correspondence criterion, a probability estimate is accurate if it coincides with the corresponding objective probability. We evaluated this type of accuracy with the root mean squared error (RMSE), the mean error (ME), and the correlation (*r*) between estimated and objective probabilities. The difference between *r* and the other two measures is that whereas RMSE and ME measure the numerical difference between estimated and objective probabilities, *r* measures how good participants are at rank ordering a set of conjunctive probabilities. The difference between RMSE and ME is that RMSE captures magnitude of deviation, whereas ME captures systematic bias.

According to the coherence criterion, a probability estimate is accurate if it coheres with the laws of probability theory. From the conjunction rule defined in Eq. 1 it follows that

(2a) $$p\left(\text{A}\right)\geq p\left(\text{A\&B}\right)$$

and

(2b) $$p\left(\text{B}\right)\geq p\left(\text{A\&B}\right)$$

The rule defined in Eqs 2a and 2b is known as the conjunction rule. A violation of the conjunction rule is known as a conjunction error. Here, we measured the coherence of conjunctive probability estimates by the prevalence of conjunction errors.

The vast majority of previous research on the estimation of conjunctive probabilities has focused on the coherence criterion (most often on whether estimated probabilities cohere with the conjunction rule). In an early study investigating constituent and conjunctive probability estimates, Wyer (1976) presented people with statements such as “persons rarely have Attribute A” and “persons usually have Attribute B” and asked them to estimate the probabilities that someone would have Attribute A [*p*(A)], Attribute B[*p*(B)], and both attributes [*p*(A&B)]. He predicted participants’ estimates of *p*(A&B) by multiplying their estimates of *p*(A) and *p*(B), as prescribed by probability theory (Eq. 1). The finding was that estimated probabilities of *p*(A&B) correlated very strongly with the predicted probabilities, although they were systematically higher. In other words, given participants’ estimated constituent probabilities, the conjunctive probabilities were overestimated (for a more recent study with similar design and related results, see Khemlani et al., 2012). Note that because Wyer (1976) compared estimates against probabilities generated by Eq. 1, and not against probabilities retrieved from the environment, this study concerned the coherence criterion. Although little is known about the robustness of Wyer’s (1976) first finding, hundreds of studies have replicated the finding of overestimation (e.g., Tversky and Kahneman, 1983; Wedell and Moro, 2008). Most of these have focused on the conjunction error, a phenomenon that has been argued to be a direct result of people’s overestimation of conjunctive probabilities (Nilsson et al., 2009). The conjunction error is one of the most frequently observed phenomena in research on judgment and decision making. It has been observed in a range of populations and tasks and it has been shown to be almost impossible to eliminate (for an extended review of the literature, see Wedell and Moro, 2008).

We know of no study that has focused solely on the correspondence of conjunctive probability estimates. There are, however, some studies which have touched the topic (e.g., Gavanski and Roskos-Ewoldsen, 1991; Juslin et al., 2011). These studies have typically used a task where participants first are told the values of two constituent probabilities and where they then are asked to assess the corresponding conjunctive probability. The common finding is that conjunctive probabilities tend to be overestimated.

Evidence suggests that it is the rule that people use to combine *p*(A) and *p*(B) into *p*(A&B) that leads them to overestimate conjunctive probabilities (Gavanski and Roskos-Ewoldsen, 1991). The exact properties of this combination rule are still debated but one idea that has received substantial empirical support is that people estimate the conjunctive probability by taking a configural weighted average of *p*(A) and *p*(B) (Fantino et al., 1997; Nilsson et al., 2009; Nilsson and Andersson, 2010; Jenny et al., under review; but for alternative suggestions, see, Wyer, 1976; Einhorn, 1985; Abelson et al., 1987). According to the configural weighted average model,

(3) $$p\left(\text{A\&B}\right)=\beta \times p\left(\text{A}\right)+\left(1-\beta \right)\times p\left(\text{B}\right),$$

when *p*(A) < *p*(B) and where β is a free weighting parameter (with 0.5 ≤ β ≤ 1; Nilsson et al., 2009). As its predicted conjunctive probabilities can never be lower than *p*(A), the configural weighted average model predicts conjunction errors, and thus systematic overestimation of conjunctive probabilities. The bound on β ensures that the lower constituent probability receives relatively more weight, which results in predictions that correlate strongly with objective conjunctive probabilities computed according to the multiplicative model (Eq. 1). Hence, as shown by Juslin et al. (2009), the configural weighted average model predicts both findings reported by Wyer (1976).

## Experiment 1

When people are exposed to environment *x* and asked to estimate the probability of observing Event A in environment *x*, high correspondences between the estimates and the objective probabilities have been found (Peterson and Beach, 1967). Furthermore, when experience with environment *x* is increased, the accuracy of people’s estimates of the probability that A occurs in environment *x* tends to increase (Beach et al., 1970). In Experiment 1, we tested whether increased experience with Events A and B also improves the accuracy of people’s estimates of *p*(A&B). Accuracy was evaluated with three measures related to the correspondence criterion (RMSE, ME, and *r*) and one measure related to the coherence criterion (the proportion of conjunction errors). In addition, to explore the psychological process underlying people’s conjunctive probability judgments, the weighted average model (Eq. 3) and the multiplicative model (Eq. 1) were used to predict the probability estimates at an individual level.

To our knowledge, the design used in Experiment 1 is unique in two important ways. (1) It is the first study where the accuracy of conjunctive probability estimates is studied in a task where the participants have to learn all task-relevant information. (2) It is the first study where both the numerical difference and the correlation between estimated and objective conjunctive probabilities are measured (please note that Wyer, 1976, did not compare against objective probabilities).

### Material and methods

#### Participants

Twenty-nine undergraduate students, 3 male and 26 female (*M*_age_ = 22.3 years), from the University of Basel volunteered to take part in the study. Most participants were psychology students who had completed at least one course in statistics. Hence, most participants had at least an elementary understanding of the basic concepts in statistics. Participants were compensated with either course credit or CHF 10 per half hour. In addition, participants had the opportunity to earn a bonus (described below).

#### Materials

On a computer screen, participants were presented with a black, a green, a blue, a red, and a yellow box. Each of the five boxes contained a proportion of *x* solid marbles (marbles of the same color as the box) and 1 – *x* striped marbles (white marbles with a colored stripe down the middle). The proportion of solid marbles (*x*) varied among the boxes (0.95, 0.80, 0.20, 0.50, and 0.95 for the black, green, blue, red, and yellow box, respectively). All five boxes were visible on the screen throughout the entire experiment. The positions of the boxes were randomly assigned to the participants.

#### Design and procedure

The experiment was a 2 × 2 within-subject design with level of experience (low vs. high) and event type (constituent vs. conjunctive) as independent variables. The experiment took about 1 h to finish and was divided into two blocks. Each block consisted of an experience phase and an estimation phase. In the experience phase, participants learned about the proportion of solid and striped marbles in each box by sampling. In the estimation phase the participants were asked to give constituent and conjunctive probability judgments.

In the beginning of the experience phase, a red arrow appeared above the (*color*) box. Then the participants were told, “Ten marbles will be sampled from the (*color*) box” and asked, “How many do you think will be solid?” Participants answered by clicking on one of 11 buttons labeled 0–10. Then 10 marbles were sampled from the box, one at a time. Each marble was shown above the box for 1 s and there was a 500 ms break between the presentations of the marbles. Finally, participants were informed that “you predicted *x*” and “*y* solid marbles were sampled.” This sampling procedure was repeated once more. After the participants had experienced two samples of 10 marbles each, they were told, “The (*color*) box includes 100 marbles,” and were asked, “What proportion of these do you think are solid?” Participants responded by typing in a value between 0 and 100. This question was asked to ensure that participants remained focused throughout the whole experience phase. These steps were repeated for all five boxes going through the boxes from left to right. After that, the whole sampling procedure was repeated. Thus, during the experience phase participants experienced four samples with 10 marbles each per box. The samples were constrained such that the proportions of solid marbles sampled from the (*color*) box in each experience phase perfectly corresponded to the proportion of solid marbles in the (*color*) box. That is, in each experience phase 38, 38, 32, 20, and 8 solid marbles were sampled from the black, yellow, green, red, and blue boxes, respectively. Hence, even at the very outset, participants had been provided with the necessary information to estimate perfectly the objective probability of sampling a colored/striped marble from each box.

After each experience phase an estimation phase of 60 trials followed. Participants were asked to evaluate 10 constituent events and 20 conjunctive events twice. The presentation order was semi-random with the constraint that all 30 events were presented once before any event was presented a second time. On constituent event trials, participants were told, “One marble will be sampled from the [*color*] box. Assess the probability that it is [*striped/solid*].” On conjunctive event trials, participants were told, “Two marbles will be sampled, one from the [*Color 1*] box and one from the [*Color 2*] box. Assess the probability that the marble from the [*Color 1*] box is [*striped/solid*] and that the marble from the [*Color 2*] box is [*striped/solid*].” Participants responded by typing in a value between 0 and 1.

Upon arrival, participants received a short booklet containing instructions, which was collected at the end of the experiment. Participants read through the whole booklet before the experimental program was started. Besides describing the procedures of the experience and estimation phases, the booklet explicitly informed participants that the boxes did not contain anything other than solid and striped marbles, that the proportion of solid marbles differed between boxes, that the proportion of solid marbles in a particular box was constant throughout the experiment, and that their goal in the experience phase was to learn about the proportions of solid and striped marbles. The instructions included an introduction to the (frequentist) concept of probability. Participants were informed that “the probability that an event will occur, such as, for example, a solid marble being drawn from the black box, is typically described as a number between 0 and 1.” Four examples described that “a probability of (0/0.28/0.76/1) indicates that if marbles were sampled from the black box an infinite number of times (none/28%/76%/all) of these would be solid.” Participants were further told that conjunctions include “a combined outcome” and that the task was to “assess the probability that this combined outcome will occur.” That is, participants were explicitly told that the task “was not to make two probability judgments.”

The instructions also included information about the payoff structure, which was implemented to incentivize participants to maximize the accuracy of their subjective probability estimates. The bonus was based on a proper scoring rule, the so-called quadratic scoring rule (Gneiting and Raftery, 2007). The closer participants’ probability estimates were to the objective probabilities, the more their probability of receiving a bonus increased.

### Results and discussion

Two participants did not proceed to the second block. Thus, the data from Block 1 are from 29 participants but the data from Block 2 are from 27 participants. On average, participants predicted the samples of 10 marbles would contain 7.4, 7.5, 7.1, 5.8, and 3.8 solid marbles in Block 1 and 8.9, 8.8, 7.3, 5.2, and 3.0 solid marbles in Block 2, for the black (0.95), yellow (0.95), green (0.80), red (0.50), and blue (0.25) boxes, respectively (proportions of solid marbles in parentheses). The average estimate regarding the constituent probability of sampling a solid (striped) marble was 0.83 (0.24), 0.82 (0.27), 0.71 (0.33), 0.53 (0.49), and 0.32 (0.67) for the black, yellow, green, red, and blue boxes, respectively. Thus, participants appeared to be sensitive to the fact that the base rates of solid and striped marbles differed between boxes.

The accuracy of participants’ probability estimates are summarized in Table 1. For clarity, error was computed by subtracting objective from estimated probability. Consequently, a negative ME indicates underestimation whereas a positive indicates overestimation. The accuracy of the constituent probability estimates increased with growing experience in terms of *r* (from 0.62 to 0.79) and RMSE (from 0.27 to 0.20) but not in terms of ME, which was already low in Block 1. Two single-sample *t* tests showed that ME was significantly higher than zero in both Block 1, *t*(28) = 3.765, *p* = 0.001, and Block 2, *t*(26) = 5.869, *p* < 0.001. Hence, constituent probabilities were systematically overestimated. One-tailed independent samples *t* tests showed that the increase in accuracy was significant both when considering RMSE, *t*(54) = 2.69, *p* = 0.005, and when considering *r*, *t*(54) = 1.88, *p* = 0.033 (one-tailed *t* tests will be used throughout this paper because of the one-sided nature of the hypothesis that experience increase accuracy). As expected, more experience with the environment led to a general increase in the accuracy of the constituent probability estimates. But did increased experience with constituent events affect the accuracy of the conjunctive probability estimates?

**Table 1:** **Accuracy of participants’ probability judgments in Experiment 1**.

Block	Constituent estimates			Conjunction estimates			
	*r*	RMSE	ME^a^	*r*	RMSE	ME^a^	CE
1	0.62 (0.39)	0.27 (0.09)	0.03 (0.05)	0.41 (0.38)	0.30 (0.09)	0.18 (0.11)	0.49 (0.28)
2	0.79 (0.29)	0.20 (0.09)	0.04 (0.04)	0.62 (0.33)	0.26 (0.11)	0.17 (0.12)	0.50 (0.31)

*All values are means with standard deviations in parentheses. Accuracy was measured by the correlation (*r*), root mean squared error (RMSE), and mean error (ME) between estimated and objective probabilities as well as the proportions of conjunction errors (CEs)*.

*^a^A positive value indicates overestimation*.

More experience substantially increased the correlation between the estimated and the objective conjunctive probabilities from 0.41 to 0.62. A one-tailed independent samples *t* test showed that this increase in *r* was significant, *t*(54) = 2.25, *p* = 0.014. The overall deviation between estimated and objective conjunctive probabilities, as measured by RMSE, decreased from Block 1 (0.30) to Block 2 (0.26), though the decrease was not statistically significant: one-tailed, *t*(54) = 1.28, *p* = 0.103. The level of ME was high, thereby indicating overestimation, and did not change from Block 1 (0.18) to Block 2(0.17). Two single-sample *t* tests revealed that the overestimation was significant in both Block 1, *t*(28) = 8.564, *p* < 0.001, and Block 2, *t*(26) = 6.949, *p* < 0.001. Thus, while increased knowledge about constituent probabilities was accompanied by an increased correlation between conjunctive probability estimates, more experience left the overestimation of conjunctive probabilities unaffected. Notably, this is the result that would be predicted if participants combined probabilities by means of the configural weighted average model (Eq. 3).

The mean proportion of conjunction errors was high in both Block 1 (0.49) and Block 2 (0.50). If conjunction errors were mainly attributable to random fluctuations in probability estimates (as argued by Costello, 2009), then one would expect the proportion of conjunction errors to be correlated with the level of consistency in conjunctive probability estimates. The correlation between the proportion of conjunction errors and the level of consistency in conjunctive probability estimates – measured by the root mean squared deviation (RMSD) between the first and second estimates within one estimation phase – was close to zero in both Block 1 (*r* = −0.002, *p* = 0.992) and Block 2 (*r* = 0.084, *p* = 0.677). This result provides evidence against the random fluctuation hypothesis.

To explore the psychological process underlying people’s conjunctive probability judgments, the weighted average model (Eq. 3) and the multiplicative model (Eq. 1) were used to predict the probability estimates at an individual level. To equate the flexibility of the models, we *a priori* fixed the weight β of the weighted average model to 0.80. We motivate this choice from an ecological rationality as well as an empirical perspective. First, a weight of this magnitude maximizes the model’s ecological validity. Computer simulations in which the weighted average model predicted conjunctive probabilities based on noisy samples have shown that the resulting conjunctive probabilities correlated highest with the true underlying conjunctive probabilities for β = 0.80, irrespective of the level of noise contained in the constituents (Juslin et al., 2009). Thus, a weight of 0.80 allowed the model to produce conjunctive probabilities that were most adapted to the environment. Second, recent empirical evidence suggests that a weight of this magnitude describes well how heavily people weight the smaller of two constituent probabilities when assessing conjunctive probabilities. Using a hierarchical Bayesian parameter estimation to fit the weighted average model to people’s behavior resulted in best fitting parameter values which fluctuated around a value of 0.80 (Jenny et al., under review). Therefore, fixing β to 0.80 makes most sense ecologically as well as empirically.

For each participant we computed the mean of the two probability estimates for each constituent item and used these to compute model predictions. Note that each item had been presented twice. We compared the predictive accuracies of the two models by the RMSD between model predictions and the mean observed conjunctive probability estimates. As the goal was to investigate the cognitive processes behind *systematic* conjunctive probability estimates, we conducted this model comparison with only those participants who provided consistent conjunctive probability estimates. More precisely, we analyzed only the data of participants with statistically significant correlations between the first and second conjunctive probability estimates within one estimation phase. Ten participants in Block 1 and seven participants in Block 2 had non-significant correlations (*r* = −0.35 to 0.43, *p* > 0.05) and were therefore excluded from this analysis.

With a mean RMSD of 0.17 (SD = 0.06) in Block 1 and 0.16 (SD = 0.04) in Block 2 the weighted average model had higher overall predictive accuracy than the multiplicative model (Block 1: *M* = 0.20, SD = 0.07; Block 2: *M* = 0.19, SD = 0.09). Fourteen of 19 participants (74%) in Block 1 and 13 of 21 participants (62%) in Block 2 were better predicted by the weighted average model. This difference between the number of people best described by the weighted average model vs. the multiplicative model was significant in Block 1, χ^2^(1) = 4.26, *p* = 0.039, but not in Block 2, χ^2^(1) = 1.19, *p* = 0.275.

**Figure 1:**
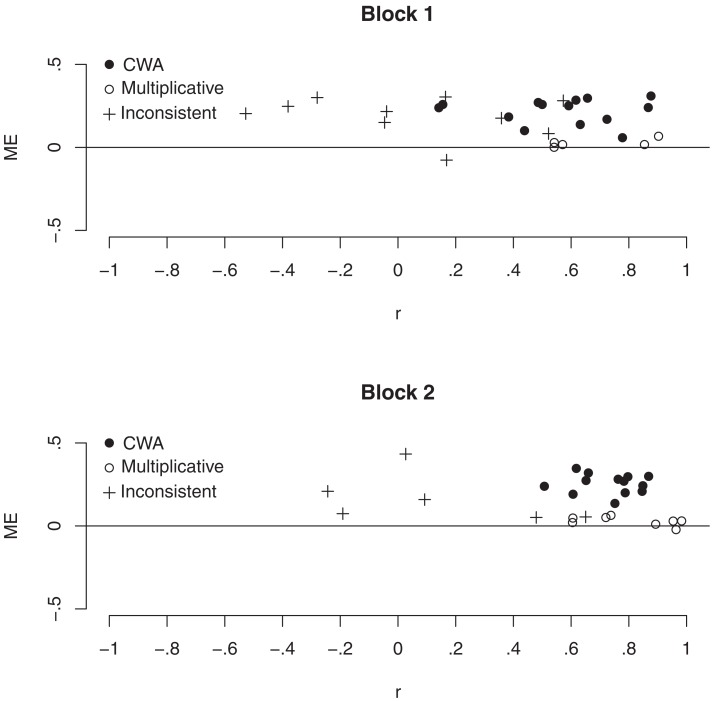
**Mean error (ME) plotted against the correlation (*r*) between estimated and objective conjunctive probabilities for each participant and block in Experiment 1**. Symbols indicate whether the participant’s data was best fit by the configural weighted average model (CWA), best fit by the multiplicative model, or excluded from the model-analysis.

In Figure 1, individual RMSE is plotted against individual *r* (between estimated and objective conjunctive probabilities). Black circles represent participants whose estimates were best predicted by the weighted average model, white circles represent participants whose estimates were best predicted by the multiplicative model, and pluses represent participants who were excluded from the model-analysis according to the above-mentioned exclusion criterion. Three groups of participants can be identified (especially clearly in Block 2). The first group performed relatively poorly on both accuracy dimensions. Incidentally, these participants also displayed low reliability in their probability estimates and were subsequently excluded from the model comparison. A second group performed well on the correlation dimension but poorly on the deviation dimension. The probability estimates of these participants were best predicted by the weighted average model. The final group performed well on both accuracy dimensions, and their estimates were best predicted by the multiplicative model. Notably, although participants whose estimates were best predicted by the weighted average model systematically overestimated objective probabilities, their probability estimates correlated just as strongly with the objective probabilities as the estimates of the participants who were best described by the multiplicative model. This matches the previous finding that conjunctive probabilities, which are based on noisy approximations of objective constituent probabilities, correlate just as well with the objective probabilities when they are generated by the weighted average model as when they are generated by the multiplicative model (Juslin et al., 2009).

In sum, increased accuracy of constituent probabilities, in terms of both RMSE and *r*, was accompanied by an increased correlation between estimated and objective conjunctive probabilities but it was not accompanied by a decreased overestimation of conjunctive probabilities. Thus, the answer to the question of whether increased accuracy of constituent probability estimates leads to increased accuracy of conjunctive probability estimates depends on which accuracy dimension is evaluated. If the correlation between estimated and objective probabilities is critical, then the answer is yes. In contrast, if the deviation between estimated and objective probabilities is critical, then the answer is no.

Although the majority of participants appeared to use a combination rule well captured by the weighted average model, there was a relatively large subgroup of participants who used a rule that was better captured by the normative multiplicative model. The percentage of participants ending up in this latter group was slightly higher in Block 2 (38%) than in Block 1 (26%). Hence, although most participants combined constituent probabilities by a rule that produces systematic overestimation of conjunctive probabilities, a minority used a rule that does not.

## Experiment 2

Experiment 1 showed that conjunctive probabilities are systematically overestimated and that the overestimation is relatively independent of the accuracy of related constituent probability estimates. Based on the results of a set of previous studies (Nilsson, 2008; Nilsson et al., 2009; Nilsson and Andersson, 2010; Jenny et al., under review) we suggest that this overestimation is caused by the way people combine constituent probabilities into conjunctive probabilities. Juslin et al. (2009) suggested that the reason why people rely on an alternative combination rule, such as the configural weighted average ruled (Eq. 3), is that people often lack the cognitive resources for executing the normative rule (Eq. 1). The suggestion by Juslin et al. (2009) is based on the assumption that the configural weighted average rule does not involve any multiplicative computations. Rather, the rule involves serial analog fractionation (Anderson, 1981), which in simplified terms, and adapted to the current context, means taking a large chunk of the smaller constituent probability and adding it to a small chunk of the larger constituent probability. Juslin et al. (2009) base their suggestion on the assumption that fractioning demands less than multiplication. If so, use of the normative rule might increase and, consequently, the overestimation of conjunctive probabilities might decrease if the task is made less demanding. Whether the overestimation of conjunctive probabilities can be reduced by making the estimation task less demanding was explored in Experiment 2.

Experiment 2 involved two conditions: the *memory-based condition* and the *online condition*. The former condition involved the estimation task used in Experiment 1, and the latter involved a less demanding task where participants had access to their own constituent probability estimates while estimating conjunctive probabilities. Consequently, whereas the participants in the memory-based condition had to both estimate and combine constituent probabilities in working memory, the participants in the online condition only had to combine constituent probabilities in working memory. Did this affect the overestimation of conjunctive probabilities?

### Material and methods

#### Participants

Forty-one undergraduate students, 12 male and 29 female (*M*_age_ = 23.4 years), from the University of Basel voluntarily took part in the study. Most participants were psychology students who had completed at least one course in statistics. Hence, most participants had at least an elementary understanding of the basic concepts in statistics. Participants were compensated with either course credit or CHF 10 per half hour plus a performance-contingent bonus.

#### Materials

The stimulus material differed from that used in Experiment 1 in three ways. The number of boxes was reduced to four (the proportions of colored marbles were 0.25, 0.85, 0.65, and 0.40 in the black, green, blue, and red box, respectively), the marbles were described as colored or white, and there were two pictures on each box, one of a colored marble and one of a white marble.

#### Design and procedure

The experiment was a 2 × 3 × 2 mixed design with information condition (online or memory-based, between subjects), level of experience (low, medium, or high, within-subject), and event type (constituent or conjunctive, within-subject) as independent variables. Participants were randomly assigned to either the memory-based (7 males and 14 females) or the online (5 males and 15 females) condition. To provide participants with three different levels of experience, the experiment was divided into three blocks. Each block included an experience phase and an estimation phase.

In the experience phase, focus was directed toward the (*color*) box and the participant was told, “Ten marbles will be sampled from the (*color*) box; how many do you think will be colored?” Participants answered by clicking one of 11 buttons labeled 0–10. Then, a sample of 10 marbles was created by sampling 10 times from a binominal distribution with *n* = 1 and *p* equal to the proportion of colored marbles in the (*color*) box and presented to the participants all at once. In contrast to Experiment 1, outcome feedback that “you predicted *x*” and “*y* colored marbles were sampled” was provided instantly. The feedback also included a box labeled “bonus” that turned red if the prediction was wrong and green if the prediction was correct (participants received CHF 0.20 for each correct prediction). This predicting and sampling phase was repeated 10 times. Then participants were asked: “If one marble is sampled from the (*color*) box, what is the probability that it is colored?” Participants responded by typing in a value between 0 and 1 and clicking on a button labeled “assess probability.” Upon clicking the button participants were informed that “You responded (*their response*), which means that you think that the probability of sampling a white marble is (*1* *- their response*).” This feedback was intended to help participants grasp the concept of probability. For the participants in the online condition, but not for the participants in the memory-based condition, their response was written on the box next to the picture of the colored marble and 1 minus the response was written next to the picture of the white marble. These values remained visible until the next time the participants were asked to assess the probability of sampling a colored marble from the same box. Then, the new responses replaced the old ones. Participants proceeded to the estimation phase after going through these steps once for every box. Thus, participants experienced 100 marbles being sampled from each box and assessed the probability of sampling a colored marble once for each box.

The experience phase differed from Experiment 1 in several important ways. First, participants witnessed samples of 10 marbles rather than individual marbles being drawn from the boxes. Second, more than twice as many marbles were sampled from each box. Third, constituent probabilities were assessed earlier, in the experience phase.

The estimation phase included 15 conjunction items of the same structure as in Experiment 1, which were presented in randomized order. The interface in the estimation phase was the same as the interface in the experience phase. That is, all participants saw pictures of the four boxes. Whereas the previously estimated constituent probabilities were written on the boxes in the online condition, nothing was written on the boxes in memory-based condition. Hence, the difference between the two conditions was that when assessing the conjunctive probabilities, the participants in the online condition, but not the participants in the memory-based condition, had access to their previously estimated constituent probabilities. The instructions were essentially the same as in Experiment 1. In addition to the bonus received in the experience phase, participants received a bonus of CHF 2 − *x*, where *x* equaled the RMSE of the conjunctive probability estimates multiplied by 2. Participants went through three blocks, each consisting of an experience and an estimation phase.

### Results and discussion

On average, participants predicted the samples of 10 marbles would contain 3.2, 4.3, 6.1, and 7.9 marbles in the memory-based condition and 2.9, 4.0, 6.5, and 8.1 marbles in the online condition, for the black (0.25), red (0.40), blue (0.65), and green (0.85) boxes, respectively (proportions of colored marbles in parentheses). The average estimate regarding the constituent probability of sampling a solid marble was 0.34, 0.44, 0.62, and 0.78 in the memory-based condition and 0.28, 0.41, 0.65, and 0.83 in the online condition for the black, red, yellow, and green boxes, respectively. Thus, like the participants in Experiment 1, the participants in Experiment 2 appeared to be sensitive to the fact that the base rates of solid and white marbles differed between boxes.

Participants’ probability estimates are summarized in Table 2. Correlations between estimated and objective probabilities were computed only for conjunctions because the participants assessed only four constituent probabilities per block. One participant’s probability estimate lacked variance as the participant responded 0.25 for all conjunctions and 0.5 for all constituents. This person’s estimates were therefore excluded from all analyses involving correlations. In the following, the data from the two conditions are first presented separately and then compared.

**Table 2:** **Accuracy of participants’ probability judgments in Experiment 2**.

Block	Constituent estimates		Conjunction estimates			
	RMSE	ME^a^	*r*	RMSE	ME^a^	CE
**MEMORY-BASED CONDITION**						
1	0.16 (0.17)	0.03 (0.08)	0.32 (0.44)	0.29 (0.11)	0.19 (0.10)	0.59 (0.27)
2	0.14 (0.12)	0.03 (0.07)	0.36 (0.42)	0.31 (0.13)	0.22 (0.13)	0.60 (0.28)
3	0.16 (0.17)	0.01 (0.11)	0.46 (0.47)	0.28 (0.13)	0.20 (0.11)	0.56 (0.30)
**ONLINE CONDITION**						
1	0.09 (0.05)	0.03 (0.04)	0.61 (0.39)	0.19 (0.08)	0.06 (0.11)	0.34 (0.33)
2	0.07 (0.04)	0.01 (0.03)	0.79 (0.23)	0.19 (0.08)	0.09 (0.13)	0.43 (0.36)
3	0.08 (0.07)	0.01 (0.04)	0.72 (0.34)	0.20 (0.10)	0.10 (0.14)	0.45 (0.43)

*All values are means with standard deviations in parentheses. Accuracy was measured by the correlation (*r*), root mean squared error (RMSE), and mean error (ME) between estimated and objective probabilities as well as the proportion of conjunction errors (CEs)*.

*^a^A positive value indicates overestimation*.

The results of the memory-based condition are summarized in the upper half of Table 2. Single-sample *t* tests, comparing ME to zero, revealed that while the conjunctive probabilities were systematically overestimated [Block 1: *t*(20) = 8.705, *p* < 0.001; Block 2: *t*(20) = 7.799, *p* < 0.001; Block 3: *t*(20) = 8.184, *p* < 0.001], the constituent probabilities were not [Block 1: *t*(20) = 1.056, *p* = 0.304; Block 2: *t*(20) = 1.334, *p* = 0.197; Block 3: *t*(20) = 0.294, *p* = 0.772]. As can be seen in Table 2, the accuracy level of the probability estimates, both constituent and conjunctive, remained relatively constant across blocks. The only dimension that indicated any increase in accuracy across blocks was the correlation between estimated and objective conjunctive probabilities. However, a one-way repeated measures ANOVA with *r* as dependent variable and Block as independent variable revealed that this change was non-significant, *F*(2, 40) = 1.414, *p* = 255. Thus, the positive effect that increased experience had on some of the accuracy measures in Experiment 1 was not found in the memory-based condition of Experiment 2. We suggests that the lack of change in accuracy was due to the fact that each experience phase in Experiment 2 involved more than twice as many samples as each experience phase in Experiment 1. That is, we suggest that the learning curve already reached asymptote in Block 1.

**Figure 2:**
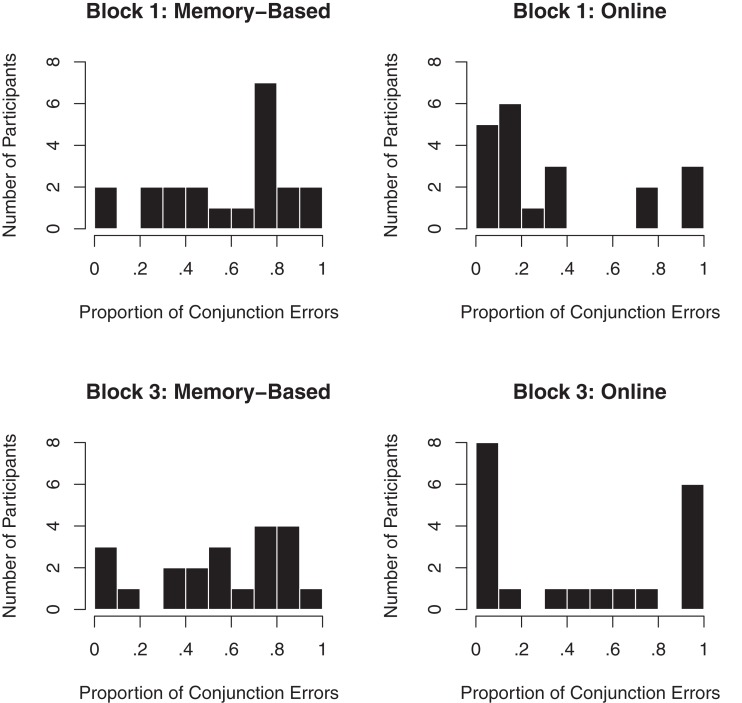
**Distributions of conjunction error rates in Experiment 2**.

The constituent probability estimates of the online condition resembled those of Experiment 1 and the memory-based condition. The levels of RMSE and ME were relatively stable across blocks. Single-sample *t* tests did not indicate any significant overestimation in any of the blocks [Block 1: *t*(19) = 1.991, *p* = 0.061; Block 2: *t*(19) = 0.115, *p* = 0.910; Block 3: *t*(19) = 0.393, *p* = 0.699]. The conjunctive probability estimates in the online condition differed substantially between individuals. This was particularly evident in data regarding the conjunction error, which is illustrated in Figure 2. In Block 1 (upper right panel), participants clustered into two groups. Some participants committed many conjunction errors but others committed few. In Block 3 (lower right panel) even clearer clusters emerged. Of the 20 participants, 8 committed conjunction errors in less than 10% of all trials and 6 committed conjunction errors in more than 90%. An additional third cluster of participants committed an intermediate number of conjunction errors. No corresponding clusters emerged in the memory-based condition (left panels in Figure 2).

In the online condition, the correlation between estimated and objective conjunctive probabilities first increased with experience from 0.61 to 0.79 but then, surprisingly, decreased again to 0.72 (Table 2). In Block 3, the correlations were bimodally distributed. The mean *r* for five participants with poor performance fell from 0.51 to 0.18 between Blocks 2 and 3, whereas for the rest of the participants, the mean *r* increased from 0.89 to 0.91. That is, although some participants were exhausted by the end of the experiment (i.e., Block 3), accuracy did increase for most participants. Notably, four of the five participants with a decreasing *r* in the last two blocks had an intermediate conjunction error rate (i.e., they belonged to the middle cluster in the lower right panel of Figure 2). Given the binominal distribution of *r*, we conducted a Friedman test with *r* as the dependent variable and block as independent variable (within-subject), which led to significant results, χ^2^(2, *n* = 19) = 7.05, *p* = 0.030. Thus, experience seemed to affect *r*. Bonferroni-corrected *post hoc* contrasts of Wilcoxon signed-ranks tests revealed that the difference between Blocks 1 and 2 was significant (*z* = 2.82, *p* < 0.05), but the difference between Blocks 1 and 3 (*z* = 1.97, *p* > 0.05) and the difference between Blocks 2 and 3 (*z* = 1.13, *p* > 0.05) were not.

The overestimation of the conjunctive probabilities was smaller than in the memory-based condition. However, single-sample *t* tests revealed that the overestimation was significant in all blocks [Block 1: *t*(19) = 2.471, *p* = 0.023; Block 2: *t*(19) = 3.178, *p* = 0.005; Block 3: *t*(19) = 3.231, *p* = 0.004]. The ME and the proportion of conjunction errors both increased across the blocks in the online condition. That is, according to these two measures, accuracy decreased across the blocks. This effect was driven heavily by data from the participants who committed conjunction errors in more than 90% of the trials in Block 3. A within-subject Friedman test with ME as dependent variable and block as independent variable showed that this decrease in accuracy was not significant, χ^2^(2, *n* = 19) = 3.36, *p* = 0.186.

As mentioned, the participants in the online condition grouped into three clusters in Block 3 (Figure 2). Did individuals in any of these groups produce more random conjunctive probability estimates than participants in the others? To answer this question, the correlation between the estimates of the conjunctive probabilities from Blocks 2 and 3 was calculated for each participant as a measure of reliability. The group that displayed the highest average reliability consisted of the participants who committed conjunction errors in more than 90% of the cases (*M* = 0.92, SD = 0.03, *n* = 6). The participants who committed conjunction errors in less than 10% also displayed good reliability (*M* = 0.78, SD = 0.32, *n* = 7). However, as indicated by the substantially larger standard deviation, the individual differences were greater in this group than in the above-90% group. Participants in the last group, who committed conjunction errors in more than 10% and less than 90% (i.e., the middle cluster in Figure 2), showed extremely low reliability (*M* = 0.14, SD = 0.38). Thus, whereas participants who committed a very small or a very large number of conjunction errors seem to have determined their conjunctive probability estimates highly systematically, the participants in the middle group seemingly showed more variance in the way they made their estimates. Notably, it was the participants in this latter group, that is, the participants committing between 10 and 90% conjunction errors, who caused the average *r* to decrease from Block 2 to Block 3 (see Table 2).

The goal of Experiment 2 was to explore whether creating a cognitively less demanding estimation task would increase the accuracy of the conjunctive probability estimates. Overall, the manipulation had a positive effect on all accuracy dimensions. Across blocks the mean correlation between estimated and objective probabilities was higher (0.71 vs 0.38) and the mean RMSE (0.20 vs. 0.29), mean ME (0.08 vs. 0.20), and mean proportion of conjunction errors (0.41 vs. 0.58) were lower in the online condition than in the memory-based condition. Mann–Whitney tests revealed that in the online condition the accuracy was significantly higher in terms of *r* (*z* = 2.48, *p* = 0.013), RMSE (*z* = 3.00, *p* = 0.003), and ME (*z* = 2.90, *p* = 0.004), but not in terms of the proportion of conjunction errors (*z* = 1.66, *p* = 0.097).

As in Experiment 1, the multiplicative model (Eq. 1) and the weighted average model (Eq. 3 with β = 0.80) were used to predict individuals’ probability estimates. To compare the two models over the whole experiment, the constituent and conjunctive probabilities were averaged over the three blocks. The weighted average model had a lower RMSD than the multiplicative model in the memory-based condition (0.18 vs. 0.25) and an identical RMSD in the online condition (0.16). The weighted average model predicted the probability estimates of 18 of 21 participants in the memory-based condition, better than the multiplicative model. A χ^2^ test showed that this difference between the number of participants best predicted by each model was significant, χ^2^(1) = 10.71, *p* = 0.001. In the online condition, the weighted average model predicted the probability estimates of 10 of 20 individuals better than the multiplicative model. One person was predicted equally well by both models in this condition.

How was the accuracy of conjunctive probability estimates affected by online access to subjective constituent probabilities? Overall, participants were aided by having online access to their own constituent probabilities on all accuracy dimensions. A closer look at the individual participants indicated that, as in Experiment 1 (see Figure 1), participants clustered into three relatively distinct groups and that the memory aid affected the participants of these groups differently. One group of participants seems to have responded randomly, as indicated by the low reliability in their probability estimates. These participants did not profit from the memory aid. A second group of participants could be characterized as users of the multiplicative model and a third group as users of the weighted average model. Both of these latter groups profited from the memory aid in that they became more consistent in the way they combined constituent probabilities. For the group best described by the multiplicative model, this resulted in increased accuracy in terms of both correlation and deviance between estimated and objective conjunctive probabilities. For the group best described by the weighted average model, on the other hand, the increased consistency resulted in increased accuracy only in terms of correlation between estimated and objective conjunctive probabilities. In this group, the rate of conjunction errors was actually higher with the memory aid than without.

## General Discussion

Our goal in the present study was to explore how people’s conjunctive probability estimates can be improved and how the cognitive processes behind such estimates can be described. Experiment 1 demonstrated that more experience with constituent events leads to more accurate constituent probability estimates, which are accompanied by higher correlations but not by lower deviations between the estimated and objective conjunctive probabilities. Conjunctive probabilities remain overestimated even at high levels of experience. By definition, conjunctive probabilities are smaller than their constituent probabilities and thus our finding that they remain overestimated echoes earlier work that showed that rare events are overestimated even at high levels of experience (Barron and Yechiam, 2009). In contrast to experience, memory aids that reduce cognitive demand can substantially improve conjunctive probability estimates, as we showed in Experiment 2.

The present study is unique in that conjunctive probability estimates were evaluated not only against the coherence criterion, as in almost all previous studies, but also against the correspondence criterion. As always, the analyses related to the coherence criterion revealed high, and stable, levels of conjunction errors. The analyses related to the correspondence criterion revealed two new findings. First, estimated conjunctive probabilities correlate strongly with corresponding objective probabilities. That is, people are able to rank-order conjunctive probabilities in an accurate fashion. Second, while constituent probability estimates were well calibrated (as they tend to be when the full-range response format is used; Juslin et al., 2000), conjunctive probability estimates were strongly biased toward overestimation. This is important not least because it shows that conjunction errors are caused by the overestimation of conjunctive probabilities.

In both experiments, participants’ behavior differed substantially. A relatively large minority of participants seemed to know and to prefer the normative product rule (Eq. 1) and followed it for the conjunctive probability estimates when cognitive load was low. In contrast, when the cognitive load was high, most of the participants made their conjunctive probability estimates in a way that was best predicted by the weighted average model. Following this model implies systematic overestimation of conjunctive probabilities.

Several previous studies have suggested that people overestimate conjunctive probabilities because they combine constituent probabilities with a weighting and averaging process (Nilsson, 2008; Nilsson et al., 2009; Nilsson and Andersson, 2010). It has further been suggested that people rely on such a process because it is adaptive in many environments (Juslin et al., 2009). The present study also puts some constraints on this weighted average hypothesis. First, people sometimes do follow the multiplicative rule. The probability that a person will follow the multiplicative rule or a weighting and averaging rule is apparently a function of the cognitive demands required in the judgment situation to perform the multiplicative rule. If people cannot apply the multiplicative rule for some reason, for example, because it requires high cognitive effort, they will follow a weighting and averaging rule.

Before discussing the implications of our results for research on the conjunction error, two limitations are acknowledged and the exact specification of the weighted average model is discussed. One limitation of our study, particularly of Experiment 1, is that the reliability of the conjunctive probability estimates was low. This low reliability was likely caused by participants losing focus on the relatively tedious task. As far as we can see, there is no reason to believe that the general conclusions would have changed if the reliability had been higher. On the contrary, there is one finding that strongly suggests that with higher reliability the results would just have been stronger. Many people relied on a rule that causes systematic overestimation of conjunctive probabilities, but others were sometimes able to rely on an unbiased rule. This picture emerged most clearly in Experiment 2, as shown in the two extreme clusters in the lower right graph of Figure 2 (online condition, Block 3). The participants in these two clusters had the most reliable conjunctive estimates in the whole study. We therefore conclude that, if anything, higher reliability would have most likely strengthened our results.

Our results are limited to situations of independent constituent events. However, we see no apparent reason why the level of dependence between constituent probabilities should affect the cognitive processes involved in combining them. In fact, research suggests that the process is independent of the dependence between constituent probabilities. For example, it has been repeatedly shown that conjunction errors occur in situations when constituents are both dependent (e.g., Tversky and Kahneman, 1983) and independent (Nilsson and Andersson, 2010). Furthermore, regardless of whether constituents are dependent or independent, if participants are asked to assess constituent and conjunctive probabilities, the rate of conjunction errors is typically in the vicinity of 0.4–0.6 (Wedell and Moro, 2008; Nilsson et al., 2009).

One could argue that by fixing β in Eq. 3 to 0.80 we tested a very specific version of the weighted average model and that versions with alternative weights may not have described our data equally well. In contrast, we argue that by specifying the model based on previous ecological (Juslin et al., 2009) and empirical (Jenny et al., under review) analyses we test a model whose specification reduces model complexity and thus the tendency to overfit.

## Implications for Research on the Conjunction Error

In their seminal paper, Tversky and Kahneman (1983) suggested that the conjunction error was caused by usage of the representativeness heuristic. The core feature of the representativeness heuristic is that the probability of outcome *x* given event *y* (e.g., that a draw from the black box results in the retrieval of a black marble) is determined by to what extent *x* resembles *P_y_*, the prototypical outcome of event *y* (Kahneman and Frederick, 2002). A prediction that follows is that if *x* = “a draw of a solid marble,” *P_y_* = “solid marble,” and *P_z_* = “solid marble,” then *p*(sampling a solid marble from box *y*) = *p*(sampling a solid marble from box *z*) even if *y* and *z* contain difference proportions of solid marbles. This prediction was not supported in the present study. On the contrary, the average estimated constituent probabilities clearly showed that participants were sensitive to the fact that the base rates of solid and non-solid marbles differed between boxes. This result suggests that the participants did not rely on the representativeness heuristic. Consequently, the result also suggests that the observed conjunction errors were caused by a process other than the representativeness heuristic. Hence, the present study can be added to the growing list of studies showing that usage of the representativeness heuristic is far from a necessity for the conjunction error to occur (e.g., Gavanski and Roskos-Ewoldsen, 1991; Nilsson, 2008).

Much research has been devoted to finding ways to make people avoid committing the conjunction error (e.g., Tversky and Kahneman, 1983; Crandall and Greenfield, 1986; Hertwig and Chase, 1998; Juslin et al., 2011). This research has shown that the trick is not to teach participants about which computations to use (Crandall and Greenfield, 1986), the trick is to simplify the necessary computations. One-way to do this is to alter the framing of the task (e.g., Hertwig and Chase, 1998; Juslin et al., 2011). Another way, as shown by the results of Experiment 2, is to provide aids that frees cognitive resources.

Two of our findings are particularly important for research on the conjunction error. First, rather than being caused by random error, the conjunction error is the result of a cognitive process that produces systematic overestimation of conjunctive probabilities. This finding contrasts with a hypothesis recently suggested by Costello (2009), namely, that people generally rely on the product rule, Eq. 1, but that noise in the assessment process causes occasional conjunction errors. This account predicts that the level of conjunction errors should be highest for participants with unreliable probability estimates. In contrast, we observed the highest levels of conjunction errors among participants with the most reliable probability estimates (in both Experiment 1 and Experiment 2).

Second, the cognitive process behind subjective conjunctive probability estimates produces estimates that correlate relatively strongly with objective probabilities. This finding has serious implications for at least one prominent explanation of the conjunction error, an explanation we refer to as the neglect hypothesis (e.g., Wolfe and Reyna, 2010). The neglect hypothesis suggests that the conjunction error occurs because people ignore, or at least fail to incorporate, information about one of the constituent events. Interestingly, the strength of the correlation between subjective and objective probabilities is dependent on which constituent probabilities that are ignored (for a discussion, see Juslin et al., 2009). The correlation will generally be much higher if the relatively likely constituent probability is systematically ignored than if the relatively unlikely constituent probability is systematically ignored. To see why, imagine that *p*(A) = 0.1, *p*(B) = 0.2, *p*(C) = 0.8, and that *p*(D) = 0.9 and that two participants are asked to judge *p*(A&C), *p*(A&D), *p*(B&C), and *p*(B&D) (objective probabilities being 0.08, 0.09, 0.16, and 0.18 for the four conjunctions). Imagine that participant 1 systematically neglects the unlikely constituent probability and estimates that *p*(A&C) = 0.8, *p*(A&D) = 0.9, *p*(B&C) = 0.8, and *p*(B&D) = 0.9. Imagine that participant 2 systematically neglects the likely constituent probability and estimates that *p*(A&C) = 0.1, *p*(A&D) = 0.1, *p*(B&C) = 0.2, and *p*(B&D) = 0.2. From this it is easy to see that while the estimates of participant 2 will correlate strongly with the objective probabilities (in this particular case *r* = 0.98), the estimates of participants 1 will not (in this particular case *r* = 0.17). Hence, for the neglect hypothesis to explain the high correlations between subjective and objective probabilities observed in this study, it has to assume that our participants systematically neglected the likely constituent probability. This is problematic for the neglect hypothesis because, as should be evident from the example above, if it is equipped with this assumption it does not predict conjunction errors. Thus, in this respect, our results provide evidence against the claim that the conjunction error occurs because people ignore, or at least fail to incorporate, information about one of the constituent events.

Finally, we would like to relate the findings from the present paper to the theories recently suggested by Busemeyer et al. (2011) and Tentori et al. (2013). As the theory suggested by Busemeyer et al. (2011) is grounded in quantum theory and because the theory suggested by Tentori et al. (2013) is grounded in the theory of inductive confirmation, both offer qualitatively new and fascinating approaches for studying the conjunction error. Interestingly, these two theories share the following prediction: conjunction errors will only occur in situations where there is a positive dependence of constituent event A on constituent event B. That is, if *p*(A) > *p*(B) then conjunction errors will only occur when *p*(B|A), the probability of B given A, is larger than *p*(B). In the present context, this would mean the following. Imagine that A equals “sampling a black marble from the black box” and B equals “sampling a blue marble from the blue box.” In this case, positive dependence would exist if the sampling of a black marble, or even the conception of such a sampling, increases the perceived likelihood of sampling a blue marble.

One of the main reasons for adopting the sampling paradigm was that constituent events are completely independent (which facilitates the analysis of how the accuracy of constituent probabilities affects the accuracy of conjunctive probabilities). However, even if *p*(A) and *p*(B) are in fact independent, it still might be that the judgment of *p*(A) affects the judgment of *p*(B). To see this, imagine that a participant is to combine highly likely constituent *p*(A) and highly unlikely constituent *p*(B) into *p*(A&B). If the participant starts by evaluating constituent event B this might create a negative mindset that results in a more negative evaluation of constituent event A (e.g., the participant might reason: *the first marble will certainly have the wrong color, and with my bad luck it is likely that also the second marble will have the wrong color*). However, if the participant starts by evaluating constituent event A this might create a positive mindset that results in a more positive evaluation of constituent event B. If the participant systematically starts by evaluating the more likely constituent, as suggested by for example Busemeyer et al. (2011), this might cause a systematic positive dependence of *p*(A) on *p*(B) which in turn could cause conjunction errors. If so, the level of conjunction errors should only decrease if the perceived dependence is reduced. This latter prediction is consistent with both the finding that the accuracy of constituent probabilities had little effect on the rate of conjunction errors [because there is no reason why the exact accuracy of *p*(A) should affect the perceived dependence] and the finding that the memory aid provided in the online condition of Experiment 2 reduced the rate of conjunction errors [because having *p*(B) clearly defined on the screen is likely to reduce the dependence of *p*(A) on *p*(B)]. The question of whether the conjunction errors observed in the present paper were caused by a perceived dependence between constituent events is a question of theoretical importance and, consequently, a question that will be explored in future research.

To conclude, our results imply that conjunction errors are caused by the rule people often use for combining constituent probabilities into conjunctive probabilities. Although the exact characteristics of this rule have to be explored further (as in Jenny et al., under review), it appears clear that it is a rule that produces conjunctive probabilities that correlate strongly with objective probabilities.

## Conflict of Interest Statement

The authors declare that the research was conducted in the absence of any commercial or financial relationships that could be construed as a potential conflict of interest.
